# CREB3 Transactivates lncRNA ZFAS1 to Promote Papillary Thyroid Carcinoma Metastasis by Modulating miR-373-3*p*/MMP3 Regulatory Axis

**DOI:** 10.1155/2021/9981683

**Published:** 2021-06-21

**Authors:** Gang Wang, Yuan Le, Liping Wei, Lian Cheng

**Affiliations:** ^1^Department of General Surgery (Thyroid Surgery), Affiliated Hospital of Southwest Medical University, Cardiovascular and Metabolic Diseases Key Laboratory of Luzhou, Luzhou, China; ^2^Affiliated Traditional Chinese Medicine Hospital of Southwest Medical University, Luzhou, China; ^3^Pediatric Surgery, The Affiliated Hospital of Southwest Medical University, Sichuan Clinical Research Center for Birth Defects, Luzhou, China

## Abstract

The incidence rate of thyroid carcinoma ranks ninth among human malignancies, and it accounts for the most frequent malignancy in endocrine-related tumors. This study aimed to investigate the role of long noncoding RNA (lncRNA) ZFAS1 in the metastasis of papillary thyroid carcinoma (PTC) and the potential molecular mechanisms. Both ZFAS1 and MMP3 were highly expressed in thyroid carcinoma and PTC cell, as measured by the q-PCR and TCGA database. In addition, ZFAS1 induced TPC-1 metastasis through inducing the epithelial–mesenchymal transition (EMT) process. Besides, ZFAS1 knockdown by siRNA induced miR-373-3*p* expression and reduced MMP3 expression, as quantified by q-PCR and Western blotting. According to the luciferase assay, both ZFAS1 and MMP3 were classified as the direct targets of miR-373-3*p*. However, MMP3 itself did not affect ZFAS1. Using the online prediction tool, CREB3 was predicted as the transcription factor (TF) of ZFAS1 that contained two binding sites on its promoter region, and CREB3 was positively correlated with ZFAS1 in thyroid carcinoma cohorts. Results from the dual-luciferase assay and ChIP-qPCR indicated that both the two binding sites were essential for the transcription of ZFAS1. In conclusion, CREB3 activated lncRNA ZFAS1 at the transcriptional level to promote PTC metastasis by modulating miR-373-3*p*/MMP3.

## 1. Introduction

In endocrine-related tumors, thyroid carcinoma is the most frequent malignancy [1], and its incidence rate has doubled over the last two decades. Based on the statistics from epidemiological studies, the incidence rate of thyroid carcinoma ranks ninth among human cancers [2]. Among thyroid carcinoma, papillary thyroid carcinoma (PTC) occupies near 80% of all types. PTC has a favorable prognosis, and the 5-year survival rate is over 95% after common treatments such as surgery and radioactive iodine therapy. However, progressive diseases may further develop due to local metastasis and tumor recurrence [3–5]. Actually, metastasis can be found in 10%–15% of PTC patients receiving treatments. Therefore, it is crucial to elucidate the potential molecular mechanisms of PTC metastasis and to provide prevention measures.

Long noncoding RNA (lncRNA) is a type of non-protein-coding RNA containing more than 200 nucleotides. An increasing number of studies have illustrated that the aberrant expression of lncRNAs can be observed in different diseases including cancer [6]. Recently, biomedical studies show that lncRNAs play important roles in PTC progression and development [7]. Notably, the lncRNA zinc finger antisense 1 (ZFAS1) has been identified to promote cancer development. In gastric cancer (GC), ZFAS1 plays an oncogenic role by interacting with EZH2 [8]. ZFAS1 is also upregulated in head and neck squamous cell carcinoma (HNSCC) as it is positively related to cancer initiation and metastasis [9]. In thyroid carcinoma, several studies identify that the downregulation of ZFAS1 can inhibit PTC progression through modulating microRNAs (miRNAs), and it can serve as a potential biomarker for predicting thyroid cancer prognosis [10–12]. However, the underlying molecular mechanism of ZFAS1 in PTC metastasis is still unclear because of the limited studies. Therefore, this study investigated the potential mechanisms of ZFAS1 in targeting related miRNAs.

## 2. Material and Method

### 2.1. Cell Culture and Treatment

TPC-1 and Nthy-ori 3–1 cell lines were purchased from Sigma-Aldrich and cultured in RPMI 1640 medium completed by fetal bovine serum (FBS, 10%, Gibco, USA), penicillin/streptomycin (2%), and amphotericin B (1%) at 37°C, 5% CO2. HEK-293 cells were obtained from ATCC and cultured in DMEM with 10% fetal bovine serum at 37°C with 5% CO2.

### 2.2. Vector Cloning

Ectopic expression of ZFAS1 was achieved by the pcDNA3.1 system (pcD-ZFAS1). The inserted ZFAS1 sequence was obtained through a polymerase chain reaction. The primers used are listed as follows: forward 5′-CGGGGGCCCAGGGTGGAG-3′; reverse 5′-TTTTGATTTAAAAGATTTTATTTTCTTTATG-3′. For miRNA target luciferase assay, a pGL3-control vector (Promega, USA) was used. ZFAS1 sequence containing wild or mutant miR-373-3*p* binding sites was cloned into pGL3-control vector verified by sequencing.

### 2.3. Transfection

Based on the instruction of Lipofectamine 2000 (Thermo Fisher, USA), 2 *μ*g plasmid and 25 nM miRNA or siRNA were used for transfection. Briefly, 5 × 10^5^ cells were seeded into a 6-well plate and cultured until the confluence reached 75%. Lipofectamine 2000 was mixed with plasmid or RNA at 1 : 1 ratio in Opti-MEM™ media (Gibco, USA) and then stayed at room temperature for 5 min. The mixture was added to a 6-well plate for incubating for 48 h. Specific siRNAs of ZFAS1 and MMP3 were designed and synthesized by GenePharma (Shanghai, China). The sequences (5′–3′) were as follows: siZFAS1: GTGCATGTGGTAGGTTAGATT; siMMP3: GAAGCAGTTTACTAAGAAA.

### 2.4. Real-Time PCR

After different treatments, total RNAs were extracted with High Pure RNA Isolation Kit (Roche, Switzerland) based on the protocol provided. Extracted RNAs were reverse-transcribed to cDNA with verso cDNA Synthesis Kit (Thermo Fisher, USA). Gene expression was analyzed by qRT-PCR performed with SYBR Premix Ex TaqII (Takara) and a LightCycler 480 system (Roche, Indianapolis, IN, USA). The relative expression levels were calculated using the 2^−ΔΔCT^ method (cycle threshold [CT]). The primers used in qRT-PCR are list in Table 1 (5′ to 3′).

### 2.5. Western Blot

The detailed procedure of western blot was described briefly here. After different treatments, cells were lysed with RIPA buffer and the lysate was quantified with the BCA kit according to manufacturer instruction. A total of 30 *μ*g protein of each group was loaded for electrophoresis in 10% SDS-PAGE gel. Then, protein was transferred onto PVDF membrane followed by blocking with 5% skim milk in TBST for 30 min. Primary antibodies MMP-3 (Abcam, USA) and *β*-actin (Wanlei, China) were diluted into 5% BSA dissolved in TBST at 1 : 1000 based on the datasheets of antibodies. PVDF membranes were incubated with diluted primary antibodies overnight at 4°C. Secondary antibodies were diluted into 5% skim milk dissolved in TBST (1 : 10000) and incubated with membranes for 1 h to 1.5 h at room temperature with gentle shaking. Enhanced chemiluminescence (ECL) system was employed for imaging.

### 2.6. Transwell Assay

Before seeding cells into an 8 *μ*m transwell chamber (Millipore, USA), different treatments on cells were performed in a 6-well plate 24 h earlier. Then, cells were seeded into an upper chamber at the density of 3 × 10^5^ cells/chamber. The upper chamber was filled with 200 *μ*l media without FBS, while the lower chamber was filled with 800 *μ*l media containing 10% FBS. Cells were incubated for 48 h and then washed twice with PBS. Cells were fixed with methanol for 30 min and then stained with 0.5% crystal violet for 15 min. After staining, they were washed with PBS 3 times and pictures were taken by microscope and imaging system (ZEISS, Germany).

### 2.7. RNA Immunoprecipitation

Cells were transfected with miR-373-3*p* mimics or negative control for 48 h. After transfection, cells were lysed by NP40 buffer. AGO2 (anti-AGO2, 1 : 50, Cell Signaling, USA) antibody or IgG was used and immunoprecipitated by 25 *μ*l protein A/G ligated beads. Trizol reagent was added to extract RNAs from the precipitant. And then, miR-373-3*p*, ZFAS1, and MMP3 were measured by q-PCR.

### 2.8. ChIP-qPCR (q-ChIP)

Q-ChIP was conducted according to the protocol provided by Auto iDeal ChIP-qPCR Kit (Diagenode, USA). CREB3 was overexpressed in HEK293 cells, and then linked chrome DNA was isolated through CREB3 antibody (ThermoFisher, USA) precipitation. Obtained chrome DNA was sonicated for 10 min to get DNA fragments with appropriate size. The expression of ZFAS1 was evaluated by q-PCR. Primers used for q-ChIP are list in Table 2.

### 2.9. Luciferase Assay

miRNA targets and transcription factor binding sites were confirmed by luciferase assay. The full length of ZFAS1 3′UTR region containing miRNA seed sequence was obtained through PCR and inserted into the pGL3-control vector. Mut Express® MultiS Fast Mutagenesis Kit (Vazyme, China) was used to introduce the mutant seed sequence into the pGL3-control vector. ZFAS1 promoter region was obtained through PCR based on a human genomic DNA template. Promoter sequence was inserted into the pBV-luc vector (Addgene, USA). Predicted transcription factor binding sites were mutated by Mut Express® MultiS Fast Mutagenesis Kit (Vazyme, China). Primers used in luciferase assay are list in Table 3.

### 2.10. Bioinformatics Analysis

ZFAS1, MMP3, and CREB3 differential expression profiles were analyzed from TCGA-THCA datasets downloaded from the National Cancer Institute's Genomic Data Commons (https://gdc.cancer.gov/). The expression profile of ZFAS1 based on individual cancer stages, expression based on nodal metastasis status, and the effect on overall survival rates of thyroid carcinoma patients were performed through analyzing downloaded RNA-seq data and clinical data of TCGA-THCA datasets. All data containing clinical information was saved. TPM (Transcripts Per Million) value was transformed to log2 value for analysis. ENCORI (http://starbase.sysu.edu.cn/) was used to analyze and predict miRNA targets. Transcription factor analysis was performed via JASPAR (http://jaspar.genereg.net/) and UCSC genome browser (https://genome.ucsc.edu/). The correlation between ZFAS1 and CREB3 was analyzed through the online bioinformatics tool GEPIA (http://gepia.cancer-pku.cn/index.html).

### 2.11. Statistics

All data are presented as mean ± SD values (at least three independent experiments were included). Analysis for experimental data was achieved by two-way analysis of variance (ANOVA) and *t*-test, in which analyses were performed using the GraphPad Prism8 software. For TCGA-THCA data analysis, the Mann-Whitney *U* test was performed. *p* values of <0.05 or <0.01 were considered statistically significantly different.

## 3. Results

### 3.1. lncRNA ZFAS1 Was Highly Expressed in Thyroid Carcinoma

**Figure 1:**
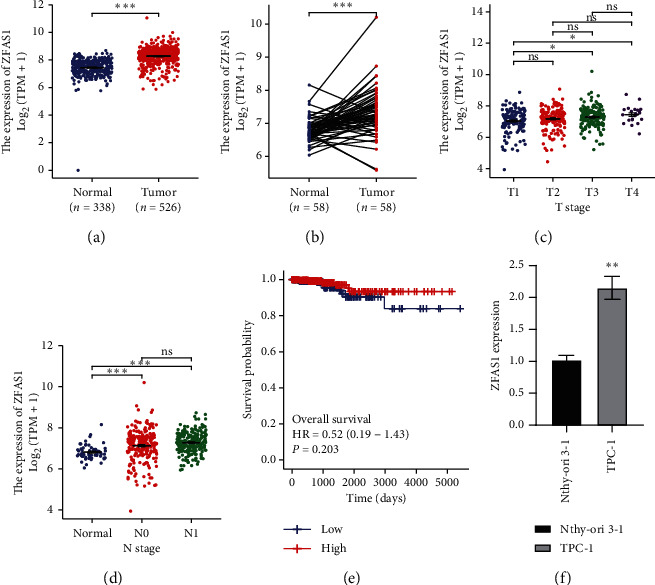
ZFAS1 expression in thyroid carcinoma. (a) ZFAS1 expression profile in primary tumor and normal tissues obtained from TCGA-THCA datasets (^*∗∗∗*^*p* < 0.001). (b) ZFAS1 expression profile in paired primary tumor and normal tissues obtained from TCGA-THCA datasets (^*∗∗∗*^*p* < 0.001). (c) ZFAS1 expression profile in different cancer stages in thyroid carcinoma obtained from TCGA-THCA datasets (^*∗*^*p* < 0.05). (d) ZFAS1 expression profile in different nodal metastasis status of thyroid carcinoma obtained from TCGA-THCA datasets (^*∗∗∗*^*p* < 0.001). (e) The relations between ZFAS1 expression and survival rate of THCA patients. The cut-off value is 50%–50% (high-low). (f) ZFAS1 expression was measured by q-PCR in TPC-1 cells and Nthy-ori- 3-1 cells (^*∗∗*^*p* < 0.01).

To obtain the ZFAS1 expression profile in clinical samples, the TCGA-THCA expression data were analyzed. As shown in Figures 1(a) and 1(b), the expression of ZFAS1 significantly increased in THCA samples compared with normal samples. In addition, ZFAS1 was also positively correlated with THCA stage (T3 and T4 stages) and node metastasis status (Figures 1(c) and 1(d)). However, ZFAS1 did not exhibit a significant effect on the survival rate (Figure 1(e)). Next, we tested the ZFAS1 expression in two different PTC cell lines by q-PCR. Indeed, in TPC-1 cells, the expression level of ZFAS1 was over 2.0 folds compared with that in human thyroid follicular epithelial cell line Nthy-ori 3–1 (Figure 1(f)). Collectively, ZFAS1 is positively related to thyroid carcinoma and potentially promotes cancer progression.

### 3.2. Suppressing lncRNA ZFAS1 Inhibited TPC-1 Metastasis

**Figure 2:**
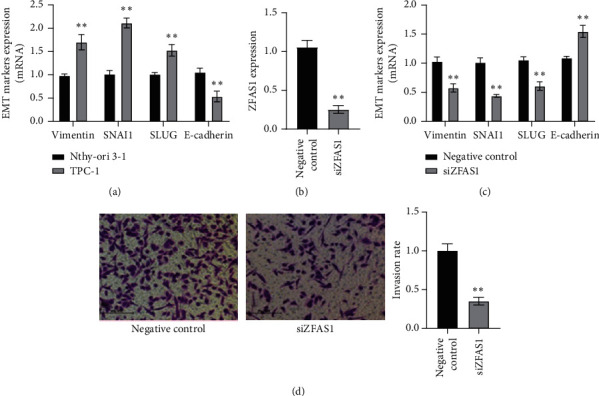
The effect of ZFAS1 on TPC-1 metastasis. (a) EMT markers were tested by q-PCR in TPC-1 cells and Nthy-ori 3–1 cells (^*∗∗*^*p* < 0.01). (b) ZFAS1 was knocked down by siRNA (^*∗∗*^*p* < 0.01). (c) The effect of ZFAS1 siRNA (siZFAS1) on EMT markers in TPC-1 cells tested by q-PCR (^*∗∗*^*p* < 0.01). (d) Invasion ability of TPC-1 cells were measured after transfected with siZFAS1 and quantified with cell numbers (^*∗∗*^*p* < 0.01).

Afterwards, the function of ZFAS1 in metastasis was evaluated. Firstly, several epithelial–mesenchymal transition (EMT) markers were tested by q-PCR. In TPC-1 cells, mesenchymal markers such as vimentin, SNAI1, and SLUG were upregulated, while epithelial markers E-cadherin were downregulated relative to Nthy-ori 3–1 cells (Figure 2(a)). Then, the specific siRNA of ZFAS1 was transfected into TPC-1 cells. ZFAS1 siRNA (siZFAS1) remarkably reduced ZFAS1 expression in TPC-1 cells (Figure 2(b)), and siZFAS1 increased the expression of E-cadherin while suppressing that of vimentin, SNAI1, and SLUG (Figure 2(c)). In addition, Transwell assay also showed that siZFAS1 inhibited the TPC-1 invasion (Figure 2(d)). The above results indicated that suppressing ZFAS1 inhibited TPC-1 cell metastasis.

### 3.3. lncRNA ZFAS1 Was a Direct Target of miR-373-3*p*

**Figure 3:**
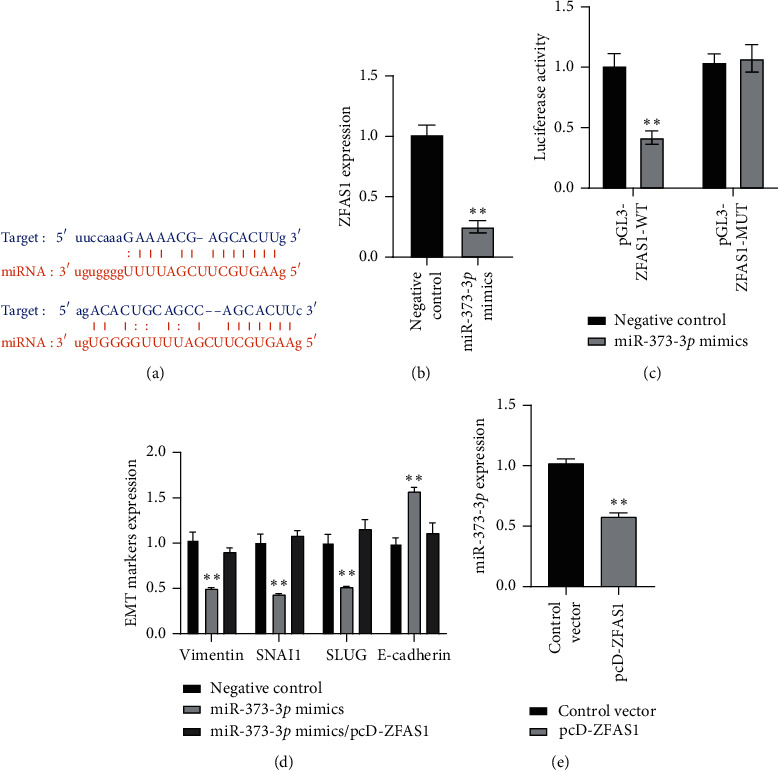
Target confirmed between ZFAS1 and miR-373-3*p*. (a) ZFAS1 was predicted as a direct target of miR-373-3*p*. (b) The effect of miR-373-3*p* on ZFAS1 expression in TPC-1 cell was tested by q-PCR (^*∗∗*^*p* < 0.01). (c) Target between ZFAS1 and miR-373-3*p* was confirmed by luciferase assay (^*∗∗*^*p* < 0.01). (d) The effect of miR-373-3*p* and miR-373-3*p*/pcD-ZFAS1 cotransfection on EMT markers in TPC-1 cells tested by q-PCR (^*∗∗*^*p* < 0.01). (e) The effect of ZFAS1 overexpression on miR-373-3*p* expression level tested by q-PCR (^*∗∗*^*p* < 0.01).

By adopting the online bioinformatic tool ENCORI, lncRNA ZFAS1 was characterized as a direct target of miR-373-3*p* (Figure 3(a)). Actually, after miR-373-3*p* mimics were transfected into TPC-1 cells, the expression of ZFAS1 was inhibited to about 20% compared with that in the negative control (Figure 3(b)). In luciferase assay, the reporter vector containing ZFAS1 wild type sequence (pGL3-ZFAS1 WT) showed decreased luciferase activity compared with the vector containing ZFAS1 mutant sequence (pGL3-ZFAS1 MUT) (Figure 3(c)). Furthermore, miR-373-3*p* also inhibited the expression of vimentin, SNAI1, and SLUG while inducing that of E-cadherin. In addition, overexpression of ZFAS1 abrogated the effects of miR-373-3*p* mimics on EMT markers (Figure 3(d)). Interestingly, the ectopic expression of ZFAS1 repressed miR-373-3*p* expression in TPC-1 cells (Figure 3(e)), indicating that ZFAS1 was not only directly targeted by miR-373-3*p* but also played a role as a miRNA “sponge” that affected miR-373-3*p* expression. The above results indicated that ZFAS1 was directly targeted by miR-373-3*p*, while ZFAS1 mediated the expression and function of miR-373-3*p*.

### 3.4. lncRNA ZFAS1 Modulated the miR-373-3*p*/MMP3 Axis

**Figure 4:**
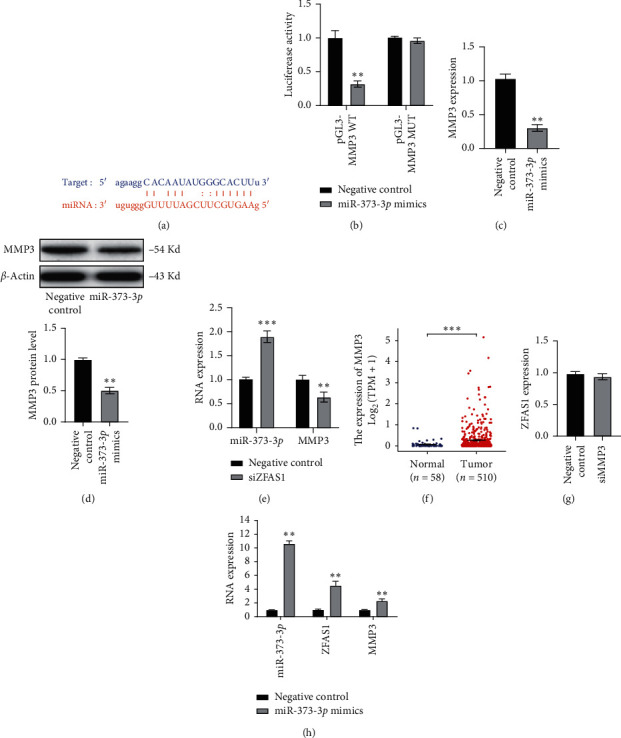
ZFAS1 modulated miR-373-3*p*/MMP3 axis. (a) MMP3 was predicted as a direct target of miR-373-3*p*. (b) Target between MMP3 and miR-373-3*p* was confirmed by luciferase assay (^*∗∗*^*p* < 0.01). (c) The effect of miR-373-3*p* on MMP3 expression in TPC-1 cell was tested by q-PCR (^*∗∗*^*p* < 0.01). (d) MMP3 protein level was decreased by miR-373-3*p* mimics (^*∗∗*^*p* < 0.01). (e) miR-373-3*p* and MMP3 expression were tested by q-PCR after ZFAS1 knocking down in TPC-1 cells (^*∗∗*^*p* < 0.01, ^*∗∗∗*^*p* < 0.001). (f) MMP3 expression profile in primary tumor and normal tissues obtained from TCGA-THCA datasets (^*∗∗∗*^*p* < 0.001). (g) The effect of MMP3 knocking down on ZFAS1 expression was tested by q-PCR. (h) ZFAS1 and MMP3 enrichment with AGO2 antibody analyzed by RNA immunoprecipitation followed q-PCR test after ectopically expressed miR-373-3*p* (^*∗∗*^*p* < 0.01).

Next, we examined whether the downstream target of miR-373-3*p* was regulated by ZFAS1. Similarly, MMP3 was predicted as a direct target of miR-373-3*p* by using the ENCORI online bioinformatics tool (Figure 4(a)). Luciferase assay confirmed that miR-373-3*p* directly targeted MMP3 (Figure 4(b)). Moreover, miR-373-3*p* mimics showed an inhibitory effect on MMP3 (Figure 4(c)). Western blotting analysis indicated that the MMP3 protein level was downregulated by miR-373-3*p* (Figure 4(d)). When siZFAS1 was transfected into TCP-1 cells, higher miR-373-3*p* expression and lower MMP3 expression were detected (Figure 4(e)). Furthermore, in TCGA-THCA datasets, it was found that MMP3 was highly expressed in primary tumor samples (Figure 4(f)). However, when MMP3 was silenced by siRNA, no significant change in ZFAS1 was observed (Figure 4(g)). At last, in RNA immunoprecipitation, both ZFAS1 and MMP3 were enriched by the anti-AGO2 antibody after miR-373-3*p* transfection (Figure 4(h)). Therefore, ZFAS1 induced MMP3 expression via the miR-373-3*p* target.

### 3.5. Transcription Factor CREB3 Activated the ZFAS1 Expression

**Figure 5:**
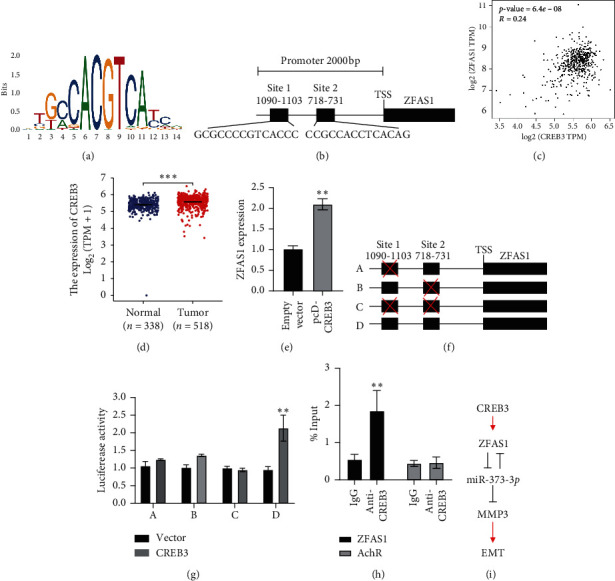
CREB3 transactivated ZFAS1 expression. (a) and (b) JASPAR predicted the positions of the putative CREB3 binding motif at −2,000 bp in the human ZFAS1 promoter. (c) CREB3 was found to be positively related to ZFAS1 expression in thyroid carcinoma. (d) CREB3 expression profile in primary tumor and normal tissues obtained from TCGA-THCA datasets (^*∗∗∗*^*p* < 0.001). (e) Ectopically expressed CREB3 induced ZFAS1 level tested by q-PCR (^*∗∗*^*p* < 0.01). (f) Luciferase reporter assay performed following cotransfection of the full-length ZFAS1 promoter or deleted ZFAS1 promoter fragments with the CREB3 expression plasmid or blank vector in TPC1 cells. (g) Luciferase activities were expressed as relative to that of the pGL3 vector (^*∗∗*^*p* < 0.01). (h) The binding between CREB3 and ZFAS1 was confirmed by q-ChIP assay; AchR was served as a negative control (^*∗∗*^*p* < 0.01). (i) Regulation model of CREB3/ZFAS1/miR-373-3*p*/MMP3 in EMT process.

There is accumulating evidence that several key TFs contribute to the dysregulation of lncRNAs in human cancers [13–15]. To analyze the potential TFs of ZFAS1, the ZFAS1 promoter region (2000 bp) was obtained from the UCSC genome browser. From JASPAR, CREB3 was predicted as a TF of ZFAS1, which contained two binding sites on its promoter region (Figures 5(a) and 5(b)). Indeed, in TCGA-THCA, CREB3 was positively correlated with ZFAS1 (Figure 5(c)). In addition, CREB3 showed higher expression in THCA samples (Figure 5(d)). CREB3 overexpression increased ZFAS1 expression in HEK293 cells (Figure 5(e)). According to dual-luciferase assay, both the two binding sites were essential for the transcription, since either the first or the second mutant site decreased luciferase activity (Figures 5(f) and 5(g)). In q-ChIP assay, compared with IgG, the use of CREB3 antibody enriched ZFAS1 after the overexpression of CREB3 (Figure 5(h)). The above results suggested that CREB3 promoted ZFAS1 expression as a TF directly binding to the ZFAS1 promoter.

## 4. Discussion

In this study, we validated that lncRNA ZFAS1 promoted thyroid carcinoma cell metastasis by regulating MMP3 via the common miR-373-3*p* targets. Furthermore, ZFAS1 was confirmed to be activated by TF CREB3, as summarized in Figure 5(i). Previous studies have shown that lncRNA ZFAS1 plays an oncogenic role by interacting with EZH2 in GC [8] and is also upregulated in HNSCC because it is positively related to cancer initiation and metastasis [9]. In line with the results found in other cancers, ZFAS1 was also confirmed to show high expression in thyroid carcinoma. Silencing ZFAS1 repressed the invasion ability of TPC-1 cells, suggesting that ZFAS1 was tightly related to thyroid carcinoma metastasis. In addition, silencing ZFAS1 induced the expression of epithelial marker E-cadherin but suppressed that of mesenchymal markers. Therefore, ZFAS1 potentially induced thyroid carcinoma metastasis through regulating the EMT process.

The competitive endogenous RNA (ceRNA) network is one of the main lncRNA regulatory mechanisms [16]. Therefore, the potential miRNA target was also investigated in this study. ZFAS1 was validated to be directly targeted by miR-373-3*p*. Actually, miR-373-3*p* was found to inhibit cancer development through targeting different lncRNAs [17, 18]. Here, miR-373-3*p* functioned as a tumor suppressor miRNA in TPC-1 cells. Of note, miR-373-3*p* inhibited ZFAS1 expression, and the ectopic expression of ZFAS1 also repressed miR-373-3*p* expression in TPC-1 cells, which indicated that ZFAS1 also functioned as a miRNA “sponge.” Furthermore, we also confirmed that miR-373-3*p* directly targeted and repressed MMP3 expression. As a member of the matrix metalloproteinase (MMP) family, MMP3 plays a key role in cell-extracellular matrix (ECM) interactions and results in EMT stimulation [19]. Thus, MMP3 is a promising downstream target of the miR-373-3*p*/ZFAS1 axis, which is possibly involved in regulating thyroid carcinoma metastasis. Notably, silencing ZFAS1 also resulted in the lower expression of MMP3, but silencing MMP3 only slightly affected ZFAS1 expression, which indicates ZFAS1 locates upstream of the regulatory axis. miR-373-3*p*, ZFAS1, and MMP3 interacted with each other through direct binding, as confirmed by RNA immunoprecipitation; therefore, the ZFAS1/miR-373-3*p*/MMP3 regulatory axis was critical in thyroid carcinoma metastasis.

In addition to the ZFAS1-related ceRNA regulation, we also investigated the potential TF as a regulator on ZFAS1. Here, TF CREB3 belonging to the bZIP family [20] was identified to activate ZFAS1 expression through directly binding to its promoter region. CREB3 belongs to the CREB3 family, which plays a key role in metabolism, tumorigenesis, and cell division. Previous studies have identified that CREB3 functions as a TF by binding to the downstream targets such as CC chemokine receptor 1 (CCR1) and Histone Deacetylase 3 (HDAC3), thus inducing tumorigenesis [21, 22]. In line with these findings, we confirmed that CREB3 and ZFAS1 were highly expressed in thyroid carcinoma. Results from both luciferase assay and q-ChIP revealed that CREB3 directly bound to the ZFAS1 promoter region. Of note, both the two putative CREB3 binding sites were essential for activating ZFAS1. Our findings indicated that lncRNA-miRNA-gene regulatory axis is critical in thyroid cancer cells which gives the hint of considering multiple targets in cancer therapy. Nevertheless, there are still limitations in this study, for instance, the lack of in vivo evidence to further support the whole CREB3/ZFAS1/miR-373-3*p*/MMP3 regulatory axis. In summary, CREB3 activated ZFAS1 drives the metastasis of thyroid carcinoma, which is involved in the miR-373-3*p*/MMP3 regulatory axis.

## Figures and Tables

**Table 1:** qRT-PCR primers.

Primers	Sequence (5′-3′)
ZFAS1-F	AAAGAGAGCGTTTCGGGTCC
ZFAS1-R	GCTCTAACGGGCAGGACAAT
Vimentin-F	GACGCCATCAACACCGAGTT
Vimentin-R	CTTTGTCGTTGGTTAGCTGGT
SNAI1-F	TCGGAAGCCTAACTACAGCGA
SNAI1-R	AGATGAGCATTGGCAGCGAG
SLUG-F	CGAACTGGACACACATACAGTG
SLUG-R	CTGAGGATCTCTGGTTGTGGT
E-cadherin-F	CGAGAGCTACACGTTCACGG
E-cadherin-R	GGGTGTCGAGGGAAAAATAGG

**Table 2:** Primers used in q-ChIP.

Primers	Sequence (5′-3′)
ZFAS1-promoter-F	ACCAGAGTGGGACGCAGGA
ZFAS1-promoter-R	TCCCCAGACCCCCATCAC

**Table 3:** Primers used in luciferase assay.

Primers	Sequence (5′-3′)
ZFAS1-F	AAAGAGAGCGTTTCGGGTCC
ZFAS1-R	GCTCTAACGGGCAGGACAAT
MMP3-F	AAGAGATATGTAGAAGGCACAATATGG
MMP3-R	TAAAATAACTGACAAATCGTCTTTATTAAAT
ZFAS1 MUT-F	AGGTCTGTCCTTTTCCTGTGCTTTCATGAAAGTGAAGATC
ZFAS1 MUT-R	CAGGAAAAGGACAGACCTTTCCAGAGGGCTCCTCTCAT
MMP3 MUT-F	GGGGTCTGTCCTTTTCTAAATGAAGCTAATAATTCTTCACCTAAGT
MMP3 MUT-R	TAGAAAAGGACAGACCCCATATTGTGCCTTCTACATATCTCTT
ZFAS1 promoter-F	ACCAGAGTGGGACGCAGGA
ZFAS1 promoter-R	TCCCCAGACCCCCATCAC
ZFAS1 promoter-MUT1-F	AGGATCAACACGATTGGGGTTATGGGGGCCTTGGG
ZFAS1 promoter-MUT1-R	CCCAATCGTGTTGATCCTTGGCGCTCCCTGACC
ZFAS1 promoter-MUT2-F	GTATCAACACGATTGGTGAGTAGGCGATGGAGGTCTG
ZFAS1 promoter-MUT2-R	CACCAATCGTGTTGATACTCAGCCCCTGGCCCAT

## Data Availability

The datasets generated during and/or analyzed during the current study are available from the corresponding author on reasonable request.

## References

[1] Xia Q., Dong S., Bian P.-D., Wang J., Li C.-J. (2016). Effects of endocrine therapy on the prognosis of elderly patients after surgery for papillary thyroid carcinoma. *European Archives of Oto-Rhino-Laryngology*.

[2] Bray F., Ferlay J., Soerjomataram I., Siegel R. L., Torre L. A., Jemal A. (2018). Global cancer statistics 2018: GLOBOCAN estimates of incidence and mortality worldwide for 36 cancers in 185 countries. *CA: A Cancer Journal for Clinicians*.

[3] Fröhlich E., Wahl R. (2014). The current role of targeted therapies to induce radioiodine uptake in thyroid cancer. *Cancer Treatment Reviews*.

[4] Haugen B. R., Alexander E. K., Bible K. C. (2016). 2015 American thyroid association management guidelines for adult patients with thyroid nodules and differentiated thyroid cancer: the American thyroid association guidelines task force on thyroid nodules and differentiated thyroid cancer. *Thyroid*.

[5] Mazzaferri E. L., Kloos R. T. (2001). Current approaches to primary therapy for papillary and follicular thyroid cancer. *The Journal of Clinical Endocrinology & Metabolism*.

[6] Iyer M. K., Niknafs Y. S., Malik R. (2015). The landscape of long noncoding RNAs in the human transcriptome. *Nature Genetics*.

[7] Tabatabaeian H., Peiling Yang S., Tay Y. (2020). Non-coding RNAs: uncharted mediators of thyroid cancer pathogenesis. *Cancers (Basel)*.

[8] Nie F., Yu X., Huang M. (2017). Long noncoding RNA ZFAS1 promotes gastric cancer cells proliferation by epigenetically repressing KLF2 and NKD2 expression. *Oncotarget*.

[9] Kolenda T., Guglas K., Kopczyńska M. (2019). Oncogenic role of ZFAS1 lncRNA in head and neck squamous cell carcinomas. *Cells*.

[10] Chen W., Zhai L., Liu H. (2021). Downregulation of lncRNA ZFAS1 inhibits the hallmarks of thyroid carcinoma via the regulation of miR3023*p* on cyclin D1. *Molecular Medicine Reports*.

[11] Han C.-G., Huang Y., Qin L. (2019). Long non-coding RNA ZFAS1 as a novel potential biomarker for predicting the prognosis of thyroid cancer. *Medical Science Monitor*.

[12] Tong H., Zhuang X., Cai J. (2019). Long noncoding RNA ZFAS1 promotes progression of papillary thyroid carcinoma by sponging miR-590-3*p* and upregulating HMGA2 expression. *OncoTargets and Therapy*.

[13] Deng X., Kong F., Li S. (2021). A KLF4/PiHL/EZH2/HMGA2 regulatory axis and its function in promoting oxaliplatin-resistance of colorectal cancer. *Cell Death & Disease*.

[14] Liang X., Lu J., Wu Z. (2021). LINC00239 interacts with c-Myc promoter-binding protein-1 (MBP-1) to promote expression of c-Myc in esophageal squamous cell carcinoma. *Molecular Cancer Research*.

[15] Zhang T., Beeharry M. K., Wang Z., Zhu Z., Li J., Li C. (2021). YY1-modulated long non-coding RNA SNHG12 promotes gastric cancer metastasis by activating the miR-218-5*p*/YWHAZ axis. *International Journal of Biological Sciences*.

[16] Landeros N., Santoro P. M., Carrasco-Avino G., Corvalan A. H. (2020). Competing endogenous RNA networks in the epithelial to mesenchymal transition in diffuse-type of gastric cancer. *Cancers (Basel)*.

[17] Wei W.-T., Wang L., Liang J.-X., Wang J.-F., Li Q., Zeng J. (2020). LncRNA EIF3J-AS1 enhanced esophageal cancer invasion via regulating AKT1 expression through sponging miR-373-3*p*. *Scientific Reports*.

[18] Zhou X.-Y., Liu H., Ding Z.-B., Xi H.-P., Wang G.-W. (2020). lncRNA SNHG16 promotes glioma tumorigenicity through miR-373/EGFR axis by activating PI3K/AKT pathway. *Genomics*.

[19] Przybylo J. A., Radisky D. C. (2007). Matrix metalloproteinase-induced epithelial-mesenchymal transition: tumor progression at snail’s pace. *The International Journal of Biochemistry & Cell Biology*.

[20] Sampieri L., Di Giusto P., Alvarez C. (2019). CREB3 transcription factors: ER-golgi stress transducers as hubs for cellular homeostasis. *Frontiers in Cell and Developmental Biology*.

[21] Jang S.-W., Kim Y. S., Lee Y. H., Ko J. (2007). Role of human LZIP in differential activation of the NF-*κ*B pathway that is induced by CCR1-dependent chemokines. *Journal of Cellular Physiology*.

[22] Kim H.-C., Choi K.-C., Choi H.-K. (2010). HDAC3 selectively represses CREB3-mediated transcription and migration of metastatic breast cancer cells. *Cellular and Molecular Life Sciences*.

